# High Order Statistics and Time-Frequency Domain to Classify Heart Sounds for Subjects under Cardiac Stress Test

**DOI:** 10.1155/2015/157825

**Published:** 2015-05-18

**Authors:** Ali Moukadem, Samuel Schmidt, Alain Dieterlen

**Affiliations:** ^1^MIPS Laboratory, University of Haute Alsace, 68093 Mulhouse, France; ^2^Department of Health Science and Technology, Aalborg University, 9220 Aalborg, Denmark

## Abstract

This paper considers the problem of classification of the first and the second heart sounds (S1 and S2) under cardiac stress test. The main objective is to classify these sounds without electrocardiogram (ECG) reference and without taking into consideration the systolic and the diastolic time intervals criterion which can become problematic and useless in several real life settings as severe tachycardia and tachyarrhythmia or in the case of subjects being under cardiac stress activity. First, the heart sounds are segmented by using a modified time-frequency based envelope. Then, to distinguish between the first and the second heart sounds, new features, named *α*_opt_, *β*, and *γ*, based on high order statistics and energy concentration measures of the Stockwell transform (S-transform) are proposed in this study. A study of the variation of the high frequency content of S1 and S2 over the HR (heart rate) is also discussed. The proposed features are validated on a database that contains 2636 S1 and S2 sounds corresponding to 62 heart signals and 8 subjects under cardiac stress test collected from healthy subjects. Results and comparisons with existing methods in the literature show a large superiority for our proposed features.

## 1. Introduction

Cardiac auscultation is the basis for heart examination. It provides a wealth of information about structural and functional cardiac defects, using a simple, efficient, and costless medical device: the stethoscope. Invented in the nineteenth century, this acoustic instrument has proved since then to be of paramount importance to the physical examination and diagnosis of cardiac pathologies. Over the course of the past two centuries, the stethoscope underwent numerous improvements to reach the development of the electronic stethoscope capable of registering and optimizing the quality of the acoustic signal, completed by the Phonocardiographic (PCG) representation of the auscultation signal. However, the analysis of the cardiac sounds, solely based on the human ear, is limited by the experience of the clinician for a reliable diagnosis of cardiac pathologies and to obtain all the qualitative and quantitative information about cardiac activity [1]. Information, such as the temporal localization of the heart sounds, the number of their internal components, their frequential content, and the significance of diastolic and systolic murmurs, can also be studied directly on the PCG signal [2]. In order to recognize and classify cardiovascular pathologies, advanced methods and techniques of signal processing will be used.

For that, two approaches could be considered to improve electronic stethoscopes:

- stethoscope with embedded autonomous analysis, simple for home use by patients and paramedics, for the purpose of autodiagnosis and follow-up,
- stethoscope coupled with a hosting device or a server for sophisticated analysis (coupled to a PC with a Bluetooth link) for the use of professionals in order to improve performance of clinical medical diagnosis.

Whatever the approach, one of the first phases in the analysis of heart sounds, is the segmentation [3–5]. Heart sound segmentation divides the PCG signal into four parts: S1 (first heart sound), extant systole, S2 (second heart sound), and extant diastole. First, S1 and S2 are located; then, extant systole is represented by the interval S1 to S2 and extant diastole by the interval S2 to S1.

Identification of the two phases of the cardiac cycle and of the heart sounds with robust differentiation between S1 and S2 even in the presence of additional heart sounds and/or murmurs is a first step in this challenge. Then, there is a need to measure accurately S1 and S2 [6, 7] allowing the progression to automatic diagnosis of heart murmurs with the distinction of ejection and regurgitation murmurs.

**Figure 1:**
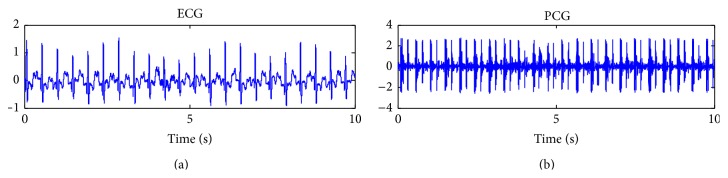
Synchronized ECG and PCG signals for a subject under cardiac stress test.

Most of the existing methods, for the direct segmentation of heart sounds, without the use of the help of ECG (see Figure 1), use the feature of systole and diastole duration to classify the first heart sound (S1) and the second heart sound (S2) [3–10]. These time intervals can become problematic and useless in several real life settings which are particularly represented by severe tachycardia, tachyarrhythmia, or subjects under cardiac stress activity.

In our earlier work on the segmentation of heart sounds [2], we applied the singular value decomposition (SVD) technique and the KNN classifier to distinguish between S1 and S2. The SVD extracts vector of 20 features is issued from the Stockwell transform [11], and then the feature vector was followed by a trained KNN classifier. This proposed method was validated on a general database (without stress data) collected from Hospital University of Strasbourg (HUS) and Mars500 project.

In this study, we use a new database of stressed subject collected in Aalborg University. This database is particularly of interest to classify S1 and S2 because it contains specific conditions where the systolic and diastolic intervals features are useless. Furthermore, our original approach adopted in this paper consists of studying qualitative features (instead of extracting blindly feature vector as done in [2]) in order to select the most appropriate single feature. This new approach makes the training phase unnecessary since it needs only a simple threshold and makes the segmentation phase less time consuming and reduces its complexity. Three original features based on time-frequency domain and high order statistics are proposed in this paper and their performances is discussed and analyzed.

The main contributions of this paper can be summarized as follows.

- A first modification on the segmentation method proposed in [2] is performed in order to enhance the detection of low intensities sounds buried in noise.
- The main contribution of this study is the investigation of 3 new qualitative features to discriminate between S1 and S2 (*α*_opt_, *β*, and *γ*):
  - the *α*_opt_ feature was used in [2] to optimize the energy concentration of the Stockwell transform. However, in this study *α*_opt_ is proposed as a feature to discriminate between S1 and S2 which is totally different and can be considered as a new approach;
  - the second feature, namely, *β*, is the integration over time of the envelope obtained by a modified measure of the instantaneous frequency of the signal. This feature aims to describe accurately the frequency content of S1 and S2 over time;
  - the third new feature, namely, *γ*, calculates the kurtosis of the time-frequency envelope. This feature is based on the spectrogram of the Stockwell transform (ST-spectrogram) and an analogy between the time-frequency coefficients and the probability density function is made in order to apply the kurtosis measure.
- Experimental validation based on specific database from Aalborg University of stress test subjects is performed. We note here that many studies in the literature suggest that an involvement of the some features extracted from the heart sounds (e.g., S/D-ratio) can increase the diagnostic value of the exercise test [12–14]. However, to our knowledge, our study is the first one with interests in distinguishing the heart sounds (S1 and S2) for subject under stress conditions.
- An experimental study to show the high frequency content ration (S2/S1) variation over heart rate is performed and discussed.

This paper is organized as follows.  Section 2 describes the data collection process and methods proposed in this paper. The results and discussion are presented in Sections 3 and 4 giving the conclusion and the future work.

## 2. Methods

### 2.1. Data Collection

The collected database used in this study corresponds to healthy subjects under cardiac stress test from the Department of Health Science and Technology, Aalborg University [15].

Nine healthy subjects were enrolled in the study (M = 5, F = 4) with a median age of 32 (24–36). Informed consent was retrieved from all subjects prior to the exercise test. A Panasonic microphone was incorporated in a coupler, specially designed by the Department of Acoustics at Aalborg University, Denmark. The microphone detects the mechanical pressure differences in the coupler, caused by alterations of the sound pressure. The microphone records with a sampling frequency of 48000 Hz. The heart sound recordings are synchronized with a 3-lead ECG (see Figure 1). The microphone was fitted to the 3rd left intercostal space with a specially designed double adhesive plaster. Subsequently the subject cycled on a Monark Ergometric 894E ergometer bicycle. The workload was increased by 25 watt every two minutes with an initial workload of 25 watt. The subject cycled until subjective maximum endurance was reached. Afterward subjects that did not reach 80% of maximum heart rate defined as (220 bmp – age) ± 12 were excluded from the study. The study was conducted according to the Danish ECG stress test guidelines. The “220 bmp – age” is a common criterion to ensure that the patient reaches their full capacity [16]. One subject did not reach this rate and was therefore excluded from the study. Recordings of heart sounds were made for 10 seconds at the end of each workload level. Acarix Data Acquisition System was used for recording the heart sounds and ECG [17]. The subject's heart rates before starting the experiment correspond to the first workload level. There were not any special restrictions required from the subjects before starting the experiment.

### 2.2. Gold Standard

The gold standard is generated based on the synchronized ECG signal. The ECG can provide information to classify the first and the second heart sounds, since S1 occurs subsequent to the QRS complex and S2 occurs after the T-wave [5].

The sounds are automatically classified based on the proposed features which are validated based on the corresponding synchronized ECG signals.

### 2.3. The High Frequency Signature (HFS) Feature to Classify S1 and S2

The only study in the literature that aims to classify S1 and S2 by taking into consideration another feature compared to the systolic and diastolic criteria is the study proposed by Kumar et al. in [18, 19]. The methods aim to extract the high frequency envelopes in sound segments, by applying the Shannon energy operator on the detail coefficients issued from the wavelet transform (Daubechies 6) [18]. In order to detect the heart cycles, an adaptive threshold is defined for this envelope. The algorithm aims to detect the high frequency signatures (HFS) and the low frequency signatures (LFS) [18].

Kumar et al. consider that usually S2 sounds contain higher frequency with respect to S1 sound (HFS correspond to S2 and LFS correspond to S1) excluding some rare exceptions.

The problem with the Kumar et al.'s paper can be summarized as follows.

**Figure 2:**
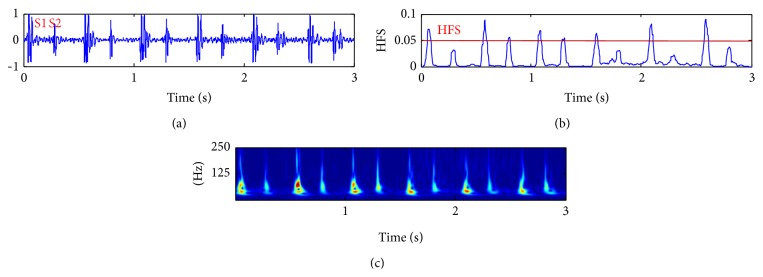
Normal heart sound with the HFS envelope and the magnitude of the S-Transform showing the higher frequency content in S1 compared to S2.

**Figure 3:**
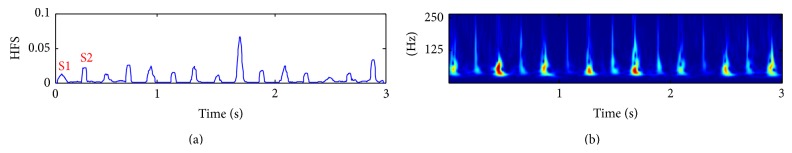
Segmented sound with the HFS envelope and the magnitude of the S-transform of the corresponding heart sound showing that HFS signature does not correspond necessarily to just one class.

- Authors consider that S1 can contain higher frequency content compared to S2 only in rare cases like prosthetic valves, for example. This hypothesis ignores the complexity of real clinical sounds on which S1 in normal sounds can have higher signature (see Figure 2, for example) and ignore that the frequency content of S1 and S2 is related to the heart rate (see Figure 15) and the auscultation position [20, 21].
- To make the proposed method automatic and free from prior knowledge, the type of HFS signature is not identified as S2 automatically (because exceptions can occur) but it is identified by using the systolic time interval criteria which is exactly what we aim to avoid in this study since the systolic time interval estimation is not a reliable feature in stress test data (when HR is high) or in pathological cases as severe tachycardia or tachyarrhythmia.
- Authors consider that all detected HFS exhibit one class of sound (S1 or S2) which is not a reliable hypothesis since the frequency contents of S1 and S2 can vary in the same registration (see Figure 3) due to changes in respiratory conditions [20, 21].

### 2.4. Stockwell Transform

The S-transform originates from two advanced signal processing tools, the short time Fourier transform (STFT) and the wavelet transform (WT). It can be viewed as a frequency dependent STFT or a phase corrected WT. The S-transform is becoming a valuable tool applied on many signals and domains as cardiovascular [2], EEG signals [22], geophysics [23], power system engineering [24], and so forth. The S-transform of a time varying signal *x*(*t*) is defined by [11]

(1) $$\begin{array}{c} S_{x}\left(t,f\right)=\overset{+\infty }{\underset{-\infty }{\int }}x\left(\tau \right)w\left(\tau -t,f\right)e^{-2\pi jf\tau }d\tau , \end{array}$$

where the window function *w*(*τ* − *t*, *f*) is chosen as

(2) $$\begin{array}{c} w\left(t,f\right)=\frac{1}{\sigma \left(f\right)\sqrt{2\pi }}e^{-t/2\sigma^{2}\left(f\right)}. \end{array}$$

And *σ*(*f*) is a function of frequency as

(3) $$\begin{array}{c} \sigma \left(f\right)=\frac{1}{\left|f\right|}. \end{array}$$

The window is normalized as

(4) $$\begin{array}{c} \overset{+\infty }{\underset{-\infty }{\int }}w\left(t,f\right)dt=1. \end{array}$$

This gives the direct relation between the S-transform and the Fourier spectrum by averaging the local spectrum over time:

(5) $$\begin{array}{c} \overset{+\infty }{\underset{-\infty }{\int }}S_{x}\left(t,f\right)dt=X\left(f\right), \end{array}$$

where *X*(*f*) is the Fourier transform of *x*(*t*).

### 2.5. Segmentation: The Modified SSE Method

The localization of heart sounds is established by using the SSE method (see 6). The proposed SSE method extracts the envelope of the signal by calculating the Shannon energy of each column of the extracted S-matrix (local spectrum). Then, the extracted envelope is smoothed by applying an average filter. The SSE envelope applied on the time-frequency matrix *S*(*τ*, *f*) is calculated as:

(6) $$\begin{array}{c} \text{SSE}\left(S\left(\tau ,f\right)\right)=-\overset{+\infty }{\underset{-\infty }{\int }}{\left|S\left(\tau ,f\right)\right|}^{n}\log\left({\left|S\left(\tau ,f\right)\right|}^{n}\right)df. \end{array}$$

**Figure 4:**
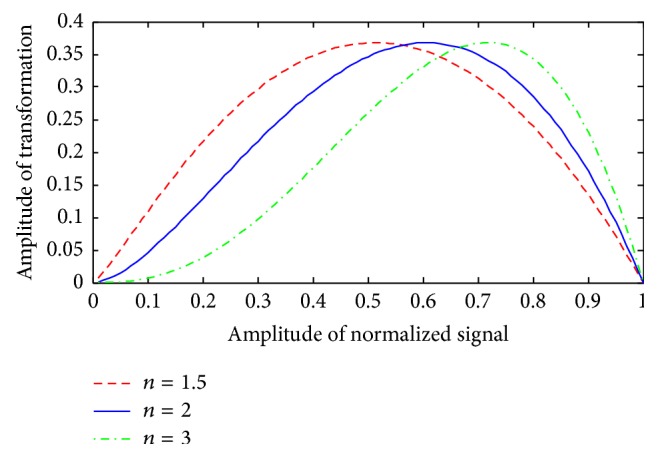
The envelope of normalized signal for values of *n* = 1.5, 2, and 3.

**Figure 5:**
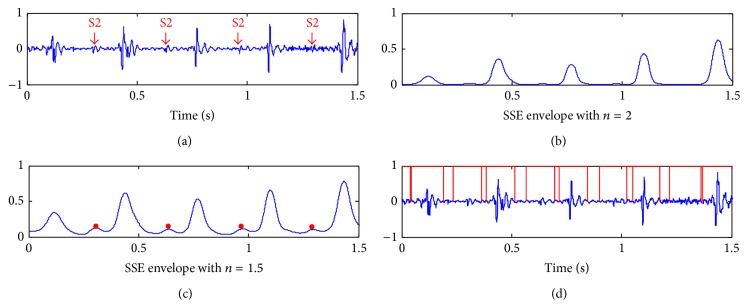
The influence of the values of *n* in the SSE envelope for the detection of S2 sounds with very low intensities.

The parameter *n* is usually fixed to 2 [2] which is the standard coefficient of the Shannon energy measure. In this study, *n* is fixed to 1.5 to enhance the detection of low intensities sounds buried in noise. This occurs in heart sounds more often with S2 when the cardiac frequency is high.  Figure 4 shows the compromise of attenuation of low and high intensities, as a function of the value of *n*. We note here that, for the SSE method, the intensities are the local spectrum coefficients of the S-transform and not the time sample intensities of the signal.  Figure 5 shows the influence of the values of *n* in the detection of very low intensities heart sounds (S2 in this case).

### 2.6. New Proposed Features

*Feature 1 (the Gaussian parameter (α*_*opt*_ *)).* In another study, we have introduced a parameter *α* to the Gaussian window in order to optimize the energy concentration of the Stockwell transform [2]. The parameter *α* introduced to the Gaussian equation 2 is introduced as follows:

(7) $$\begin{array}{c} \sigma \left(f\right)=\frac{\alpha }{\left|f\right|}. \end{array}$$

The value of *α* which maximizes the energy concentration is considered as the optimal value. The energy concentration measure is given as

(8) $$\begin{array}{c} \text{CM}\left(\alpha \right)=\frac{1}{\int_{-\infty }^{+\infty }\int_{-\infty }^{+\infty }\left|\overset{-}{S_{x}^{\alpha }\left(t,f\right)}\right|dt\;df}. \end{array}$$

This measure has some favorable performance in comparison to other concentration measures [25].

With $\bar{S_{x}^{\alpha }(t,f)}$ being the normalized energy of the S-transform for each *α*, it is given by [26]

(9) $$\begin{array}{c} \bar{S_{x}^{\alpha }\left(t,f\right)}=\frac{S_{x}^{\alpha }\left(t,f\right)}{\sqrt{\int_{-\infty }^{+\infty }\int_{-\infty }^{+\infty }{\left|S_{x}^{\alpha }\left(t,f\right)\right|}^{2}dt\;df}}. \end{array}$$

The values of *α* are chosen from a set, 0.5 < *α* < 2, with a step of 0.1. The optimal solution is reached when CM(*α*) is maximized:

(10) $$\begin{array}{c} \alpha_{\text{opt}}=\underset{\alpha }{\arg\;\max}\left(\text{CM}\left(\alpha \right)\right). \end{array}$$

In this study, we propose to test the ability of the *α*_opt_ to discriminate between the first and the second heart sound. Since it has been used to optimize the energy concentration in the time-frequency plane, it may be interesting to test it as a discriminator feature. From a signal theory point of view, the complexity concept of signals is intuitively related to the number of their elementary components [27] and since S1 generally contains more components than S2 [21], hence, it can be considered as a more complex signal than S2. These physiological differences will necessarily lead to different time-frequency content behavior which we will aim to reveal with *α*_opt_ parameter.

**Figure 6:**
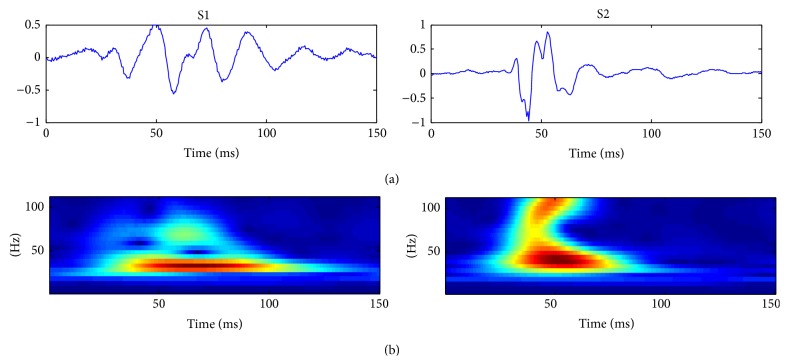
S1 and S2 signals (a) and optimized S-transform obtained with *α* = 0.8 for S1 and *α* = 0.5 for S2 (b).

Figure 6 shows S1 and S2 signals examples with the corresponding optimized S-transform obtained with *α* = 0.8 and 0.5, respectively.

*Feature 2 (the SSE envelope feature (β)).* It is another new feature that we investigate in this study, namely, *β*; it aims to integrate the normalized SSE envelope over time; it can be given as

(11) $$\begin{array}{c} \beta =\overset{+\infty }{\underset{-\infty }{\int }}\left\{\overset{+\infty }{\underset{-\infty }{\int }}{\left|S_{x}\left(t,f\right)\right|}^{2}\text{lo}{\text{g}}_{2}\left({\left|S_{x}\left(t,f\right)\right|}^{2}\right)df\right\}dt. \end{array}$$

**Figure 7:**
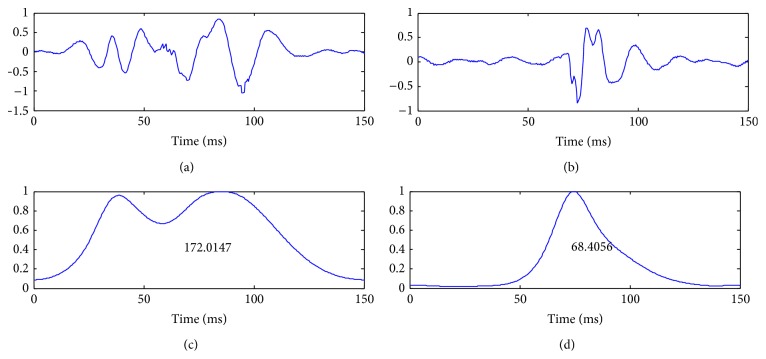
S1 (left) and S2 (right) signals and their normalized SSE envelopes with the values of *β* (bottom).

The SSE envelope estimates the frequency energy at the local spectrum of the signal. It can be considered as a modified instantaneous frequency measure. The *β* feature aims to reveal the frequency contribution of each sound over time. Mathematically, it can be viewed as the integration over time of a modified instantaneous frequency measure. The measure is computed from the normalized SSE envelope to avoid the influence of the amplitude variations.  Figure 7 shows an example of the *β* feature calculated on S1 and S2 sounds from their normalized SSE envelopes.

*Feature 3 (high order statistic feature (γ)).* It is the third feature proposed in this paper on higher order statistic measure (kurtosis) applied on time-frequency coefficients. The kurtosis measure is normally applied on a probability distribution to describe its shape. A normal transition between the Stockwell transform and the corresponding time-frequency energy distribution is the square of magnitude of the S-matrix, namely, in this paper, the ST-spectrogram. In this case, the time-frequency representation plays an analogous role to a 2D probability density function (PDF) [27]. Then, the kurtosis can be applied directly on this estimated PDF via time-frequency plane. However, the kurtosis may be very sensitive to noise [28]. To deal with this problem, we calculate the SSE envelope applied on the ST-spectrogram before applying the kurtosis. In this case, the values of the extracted envelope are considered as the estimated probability distribution via time-frequency plane.

If we consider the squared modulus of the S-transform or the ST-spectrogram, we obtain an energy distribution of the signal in time-frequency plane. The ST-spectrogram is given as

(12) $$\begin{array}{c} {\left|S_{x}\left(t,f\right)\right|}^{2}={\left|\overset{+\infty }{\underset{-\infty }{\int }}x\left(\tau \right)w\left(\tau -t\right)e^{-2\pi jf\tau }d\tau \right|}^{2}. \end{array}$$

The ST-spectrogram is normalized as follows:

(13) $$\begin{array}{c} {\left|S_{x}^{\mathrm{norm}}\left(t,f\right)\right|}^{2}=\frac{{\left|S_{x}^{\mathrm{norm}}\left(t,f\right)\right|}^{2}}{\iint {\left|S_{x}^{\mathrm{norm}}\left(t,f\right)\right|}^{2}dt\;df}. \end{array}$$

The proposed feature based on the kurtosis can be given as

(14) $$\begin{array}{c} \gamma =\frac{E\left[{\left(\text{SSE}\left({\left|S_{x}^{\mathrm{norm}}\left(t,f\right)\right|}^{2}\right)-\mu_{x}\right)}^{4}\right]}{{\left\{E\left[{\left(\text{SSE}\left({\left|S_{x}^{\mathrm{norm}}\left(t,f\right)\right|}^{2}\right)-\mu_{x}\right)}^{2}\right]\right\}}^{2}}, \end{array}$$

where SSE(|*S*_*x*_^norm⁡^(*t*, *f*)|^2^) are the values of the SSE envelope applied on the normalized ST-spectrogram |*S*_*x*_^norm⁡^(*t*, *f*)|^2^ and *μ*_*x*_ is the corresponding mean value. The kurtosis measures the peakedness of the distribution. This feature will try to describe the shape of the estimated time-frequency distribution for the segmented sounds (S1 or S2).

**Figure 8:**
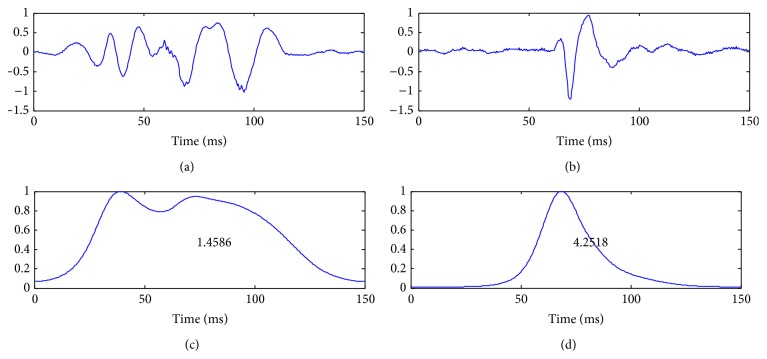
S1 (left) and S2 (right) signals and their normalized SSE envelopes with the values of *γ* (bottom).

Figure 8 shows an example of the *γ* feature calculated on S1 and S2 sounds from their SSE envelopes based on ST-Spectrogram.

## 3. Results and Discussions

The segmentation of heart sounds is established by using the modified SSE method proposed in Section 2.5. The different proposed features are tested separately and a comparison study with the HFS feature proposed in the literature is performed. The proposed features *α*_opt_, *β*, and *γ* are calculated for each segmented sound and the results are summarized in Table 1. The total number of S1 and S2 in the database is 2636 (1318 S1 and 1318 S2) sounds that correspond to 62 heart signals and 8 subjects.

### 3.1. Results for Feature *α*_opt_

Results show that the mean value of *α*_opt_ is greater for S1 than S2 (0.83 ± 0.13 and 0.59 ± 0.04, resp.), which means that the width of the Gaussian window (see Figure 5) obtained with the optimization of the energy concentration is wider for S1 than S2. In other words, the algorithm needs a higher frequency resolution for S1 than S2. The first heart sound has a booming quality and is lower pitched, duller, and longer than the second heart sound [19]; the S1 can be considered more complex (containing more components) than S2 from a physiological point of view and in term of frequency components which explain the need of higher frequency resolution or larger analysis window for S1 compared to S2.

**Figure 9:**
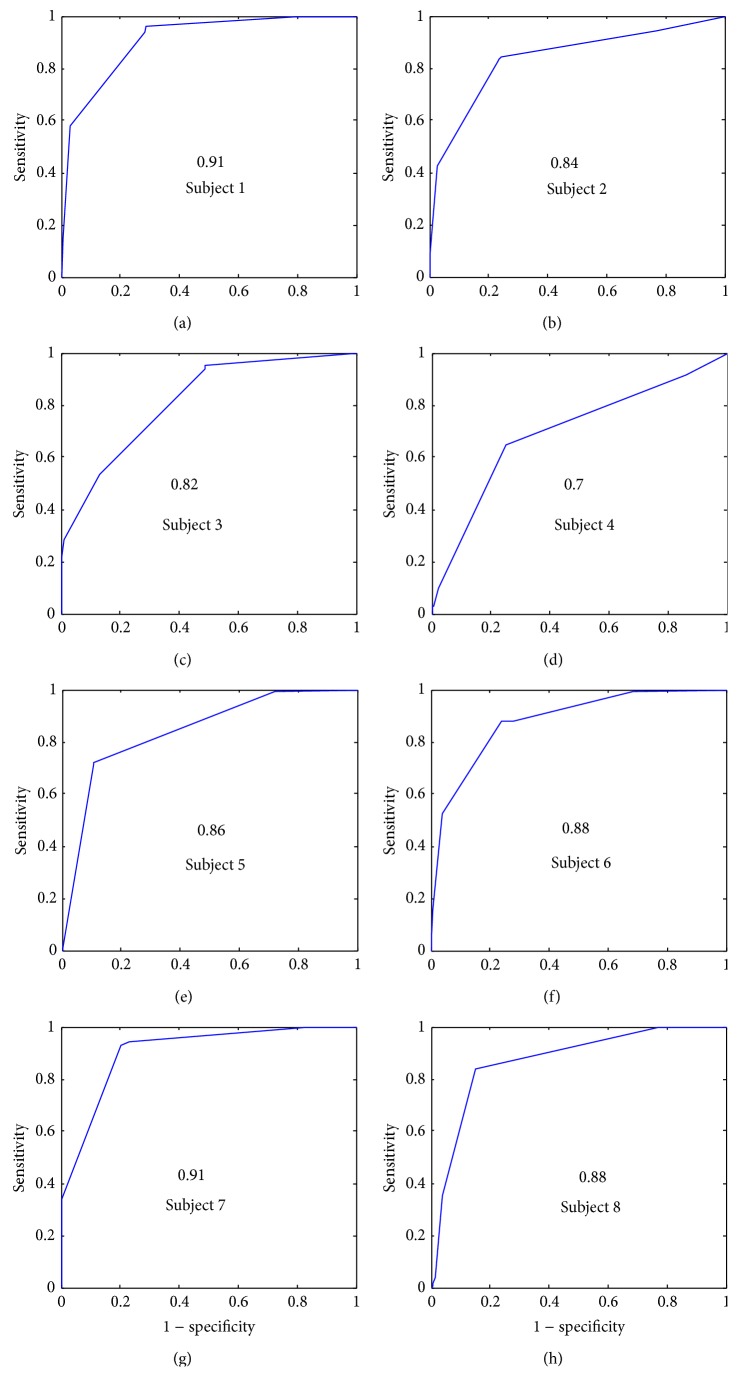
Receiver operation characteristic curves for feature *α* and for all subjects.

Figure 9 shows the AUC for the *α*_opt_ feature and for each subject. The lowest AUC corresponds to the subject 4 (0.7). The highest AUC is 0.91 and the total average of AUCs is 0.85.

The probability that the two groups (S1(*α*) and S2(*α*)) come from distributions with different medians is calculated by the Mann-Whitney *U* test (*P* < 0.0001) (Table 2). Significant differences between the two groups, with 95% confidence, are found. The classification results are promising. This is very interesting since this parameter *α* was also used to refine the boundaries detection of S1 and S2 in the segmentation process.

### 3.2. Results for Feature *β*

Results for *β* feature show that the mean value of *β* is greater for S1 than S2 (0.62 ± 0.03 and 0.34 ± 0.05, resp.). This feature is the result of the integration over time of the SSE. The SSE envelope resumes the frequency content over time; it can be viewed as an instantaneous frequency measure followed by a nonlinear filter to attenuate the low and the high frequency intensities. Hence, the *β* feature can be considered as an integration of the modified instantaneous frequency measure which will be higher for physiologically richer signals (S1 in this case).

The probability that the two groups (S1(*β*) and S2(*β*)) come from distributions with different medians is calculated by the Mann-Whitney *U* test (*P* < 0.0001) (Table 2). Significant differences between the two groups, with 95% confidence, are found.

**Figure 10:**
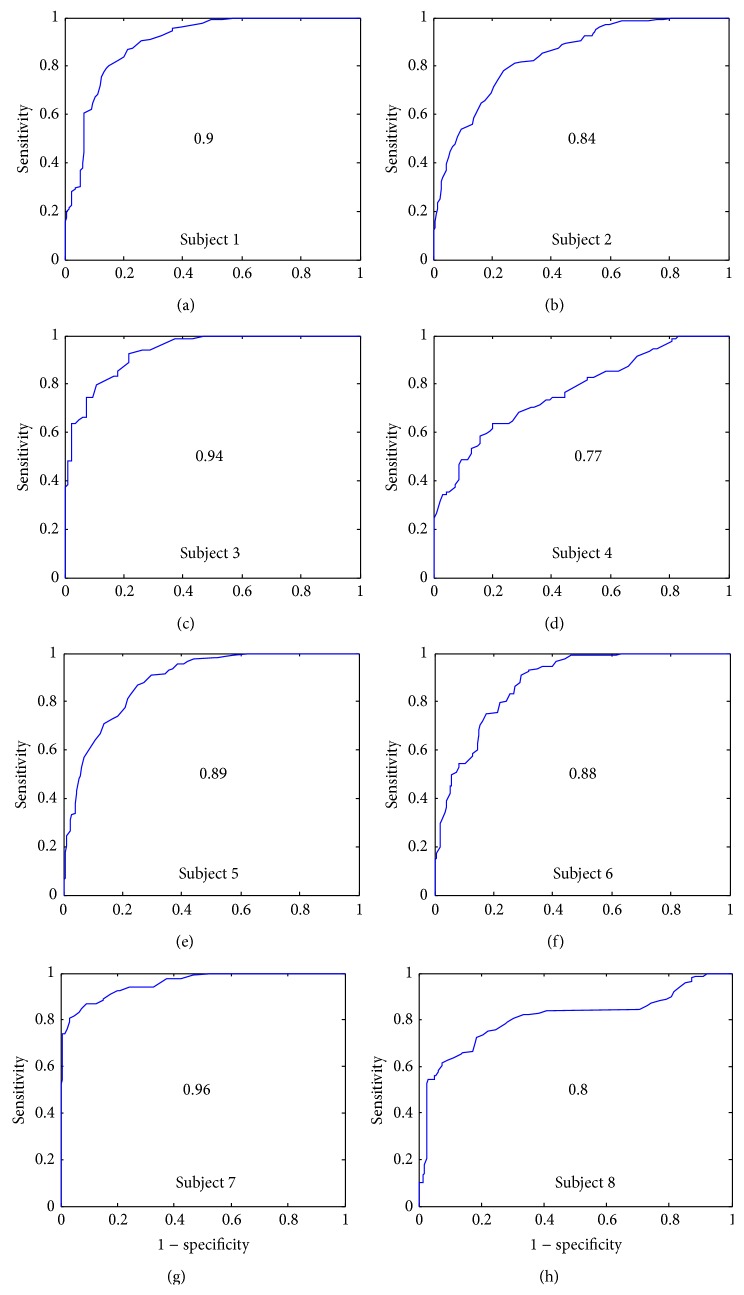
Receiver operation characteristic curves for feature *β* and for all subjects.

Figure 10 shows the AUC for the *β* feature and for each subject. The lowest AUC corresponds to the subject 4 (0.77). The highest AUC is 0.96 and the total average is 0.87 which is higher than the *α*_opt_ feature. The low AUC results for subject 4 obtained with *α*_opt_ and *β* can be explained by the high noise level in the acquired signal due to the acquisition process.

### 3.3. Results for the Feature *γ*

Results for *γ* feature show that the mean value of *γ* is greater for S2 than S1 (7 ± 2.5 and 2.89 ± 1.07, resp.). The *γ* feature operates on the distribution extracted via the spectrogram of the Stockwell transform (ST-spectrogram) which can be viewed as probability density function. The objective is to find a robust statistical description allowing us to discriminate accurately between the first and the second heart sounds.

**Figure 11:**
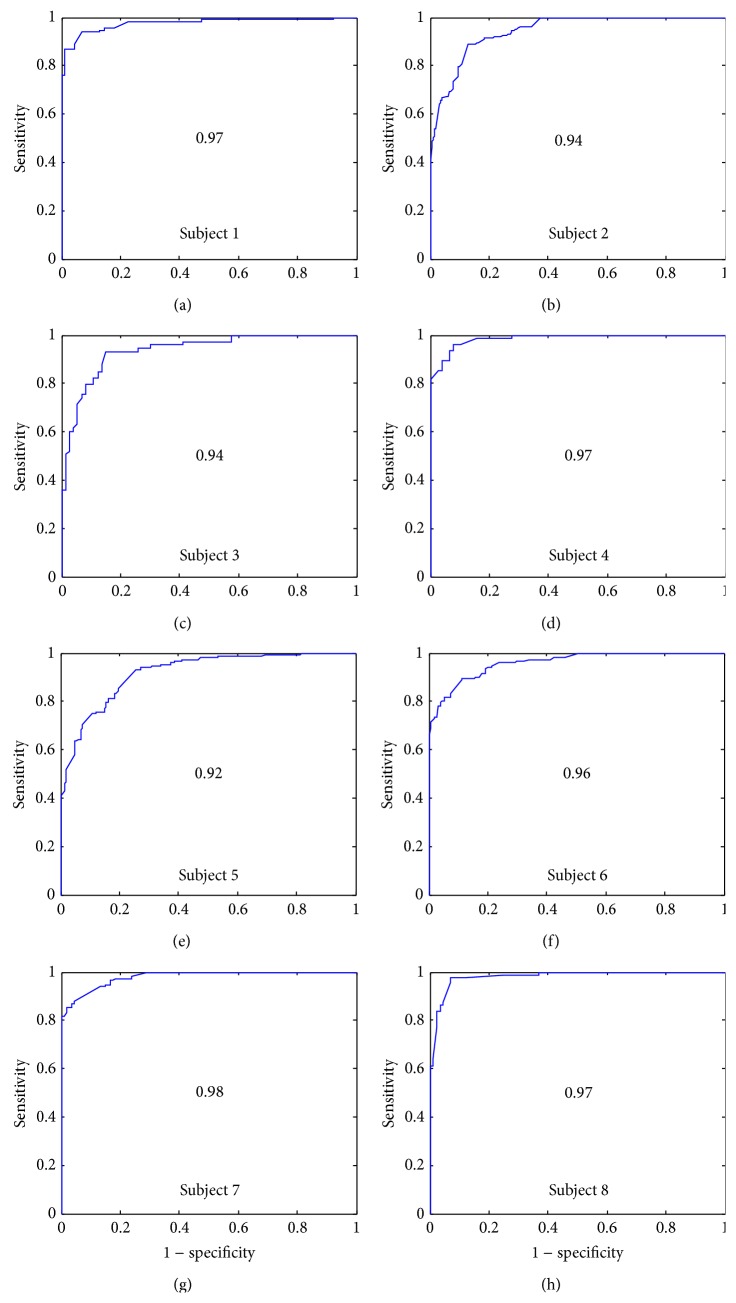
Receiver operation characteristic curves for feature *γ* and for all subjects.

The S2 distribution is a heavier tail and a higher peak than the S1 distributions. This can be explained by the fact that S1 sounds are generally longer than S2 in time and they have lower frequency signature. This will lead to higher kurtosis estimation for S2. The results for the feature showed very good performance for all subjects (Figure 11) with 0.96 of total average (AUC). The probability that the two groups (S1(*γ*) and S2(*γ*)) come from distributions with different medians is calculated by the Mann-Whitney *U* test (*P* < 0.0001) (Table 2). Significant differences between the two groups, with 95% confidence, are found.

**Figure 12:**
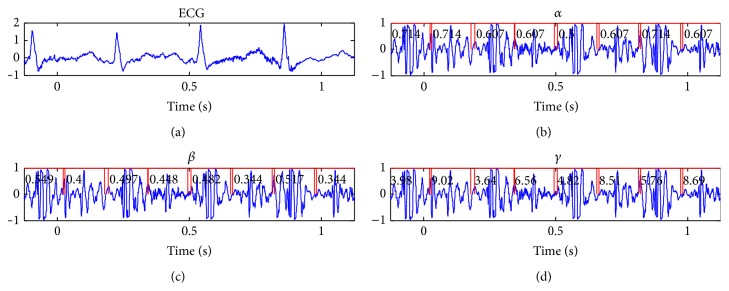
Example of a segmented stress test heart sound for subject 4 and workload level 6 (HR = 181 bmp) with the values of *α*, *β*, and *γ* calculated for each located sound (S1 and S2).

Figure 12 shows the results of segmented sound corresponding to subject 4 at the workload number 6 with a HR = 181 bmp and with different *α*, *β*, and *γ* features.

### 3.4. Comparison with the HFS Feature

**Figure 13:**
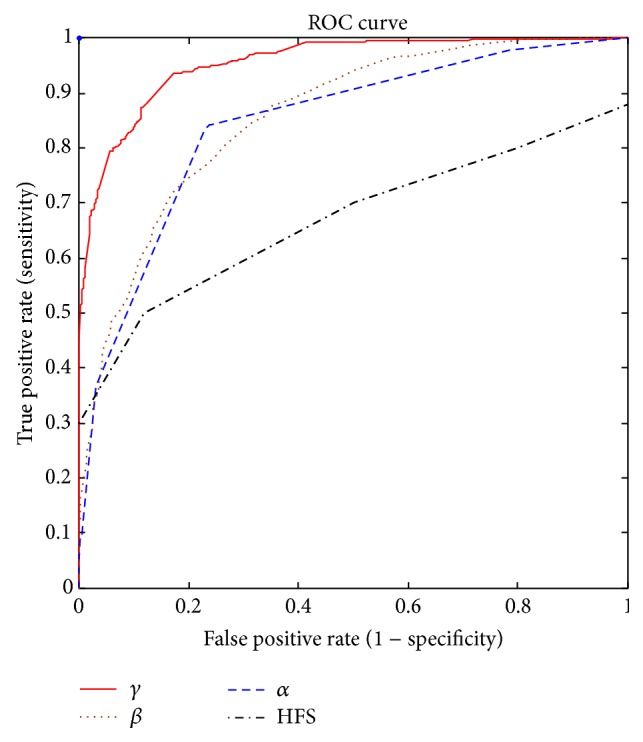
Global receiver operation characteristic curves for *α* (Feature 1, AUC = 0.85), *β* (Feature 2, AUC = 0.87), *γ* (Feature 3, AUC = 0.96), and HFS (AUC = 0.6) features.

The HFS feature shows lower results with 0.6 AUC (Figure 13). This is not surprising because the HFS method is based on several imprecise hypotheses. First, as we mentioned it above, not all HFS signatures correspond necessarily to class (S1 or S2) as authors propose in [18]. Figure 3 presents a normal heart sound with the corresponding HFS which shows clearly that the HFS does not correspond necessarily to one class. Moreover, the HFS method still needs the systolic duration to classify S1 and S2 which is not reliable when HR is very high (stress test for example). Finally, the HFS method explores only the frequency content of the sounds without any information on time; this becomes problematic for the nonstationary signals as the case in this study with S1 and S2 sounds (where the frequency of the signal varies over time). Figure 13 shows that the highest performance is reached by the proposed *γ* feature with AUC = 0.96.

### 3.5. Robustness of the Proposed Features against Noise

In this subsection, we study the robustness of the proposed features against noise. The sounds collected in the database were already contaminated with both physiological noise and background noise. Here, we will study clearly the robustness against noise of the proposed features by estimating the SNR ratio on selected heart sounds from the database and we add additive white Gaussian noise with three different levels. The average of the SNR for all sounds in the database is estimated to be 10 dB. To test the robustness against noise, two other levels of noise are added and the results are showed in Table 3.

**Figure 14:**
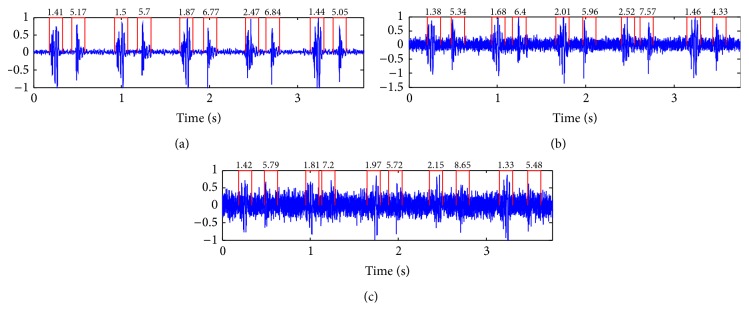
Example of a segmented stress test heart sounds with three different SNR ratios (12, 5, and 0 dB) with the values of *γ* calculated for each located sound (S1 and S2).

Results in Table 3 show clearly the high robustness of the proposed features against noise. This is not surprising since these features are based on time-frequency domain [2, 29]. The HFS feature does not show reliable results on the sounds used in this study.  Figure 14 shows a selected sound (from subject 1) with three different levels of noise and the corresponding *γ* features being calculated on the segmented S1 and S2 sounds.

### 3.6. High Frequency Content Ratio S2/S1 Variation over Heart Rate

This section aims to analyze the frequency content of S1 and S2 over the HR. As we mentioned it before, the fact that the frequency content of S2 exhibits higher frequency content than S1 cannot be generalized and adopted as robust feature to characterize the physiology related to the heart sounds. It is known, for example, that the intensity and frequency of S1 are affected by the velocity of the forces responsible for the acceleration and deceleration of the blood masses, which on the other hand are directly related to the HR [20].

To show that experimentally, the high frequency content of S1 and S2 for the 8 subjects of the database is estimated. Then, the mean and the standard deviation of the ratio of the frequency content of S2 over the frequency content of S1 (S2/S1) are calculated in relation with the heart rate. For each subject, 4 sounds are considered at 4 different stress levels (workload levels).

**Figure 15:**
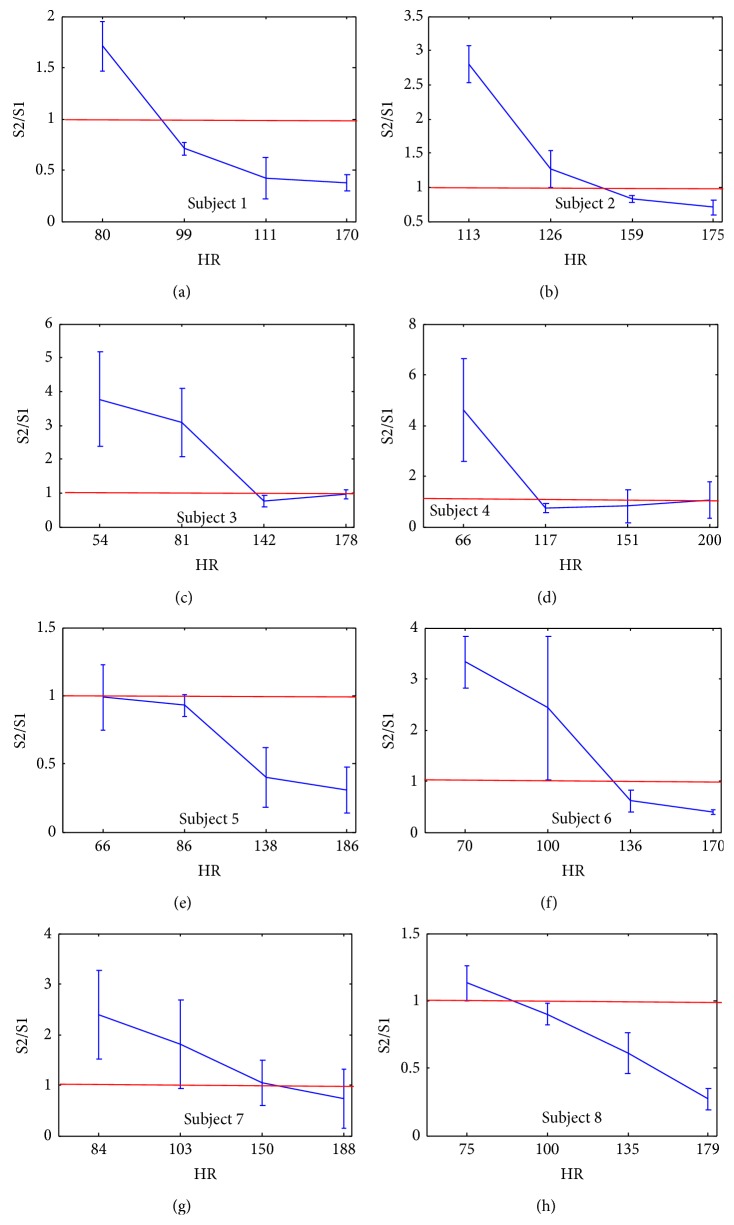
The variation of the high frequency content ratio (S2/S1) over the HR for all subjects. The red lines indicate the high frequency content of S2 becoming lower than that of S1.

**Figure 16:**
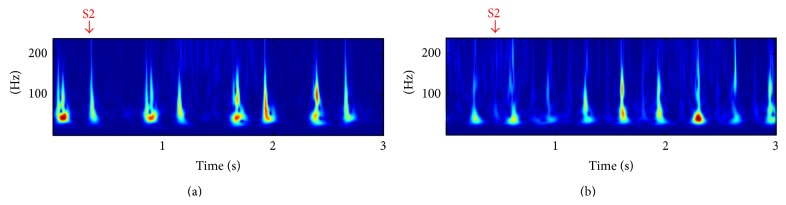
The S-transform of two sounds corresponding to the same subject and two different heart rates ((a) HR = 80 bpm, (b) 142 bpm)) showing the high frequency content of S2 decreasing when the heart rate is higher.

The results in Figure 15 show clearly the direct relation of the frequency content of the heart sounds and the heart rate. Normally, S2 has a higher frequency content than S1 (S2/S1 > 1) except for subject 5. The red line in Figure 15 indicates when the frequency content of S1 exceeds the frequency content of S2. When the heart rate increases, the high frequency content of S2 decreases which decrease the ratio S2/S1 (see Figures 15 and 16). This confirms our motivation to propose other features than the high frequency content signature to discriminate between S1 and S2.

## 4. Conclusions and Future Work

The main objective of this paper is to study the ability of new features to segment and discriminate S1 and S2 in stress test conditions data. First, we have proposed a modified version of the SSE segmentation method to take into consideration the very low intensities sounds which can occurs more often with S2 when the cardiac frequency is high. Then, we have investigated three new time-frequency features computed from the S-transform which can be considered as a hybrid method between the STFT and the wavelets. The proposed features are validated on a database that contains 2636 S1 and S2 sounds (1318 S1 and 1318 S2) that correspond to 62 heart signals and 8 subjects under cardiac stress test collected from healthy subjects.

Classifying S1 and S2 based on the PCG signal without any other reference is a hard task since they are very sensitive to several parameters like breathing, cardiac frequency, and other biomedical and environmental conditions.

The proposed features aim to describe the time-frequency behavior of each sound. The *α*_opt_ feature corresponds to the optimal width of the Gaussian window that maximizes the energy concentration of the signal. The *β* feature is the result of integration over time of the SSE envelope (which can be viewed as a modified measure of instantaneous frequency), while the *γ* feature is based on the kurtosis of the extracted SSE envelope via the normalized ST-spectrogram. The *γ* shows the highest performance with AUC = 0.96. This is very interesting since it shows the ability to discriminate accurately between clinical S1 and S2 sounds by using a single feature which simplifies the segmentation module.

The comparison with the existing method, namely, HFS (high frequency signature), in the literature [18, 19] shows a large superiority for our proposed features, most notably the *γ* feature.

Moreover, an experimental validation is performed in this study to show the high frequency content ratio (S2/S1) variation over heart rate.

Finally, the proposed methods might have high potential to study changes in the shape of the hearts sound due to splits between M/T and A/P components in the respiration cycle or to classify other biomedical and nonstationary signals.

## Figures and Tables

**Table 1:** The area under curve (AUC), mean values and standard deviations of each feature (S1(*α*), S2(*α*), S1(*β*), S2(*β*), S1(*γ*), and S2(*γ*)), and the maximum heart rate (HR_max⁡_) reached for each subject.

Subject	1	2	3	4	5	6	7	8	Mean
AUC(*α*)	0.91	0.84	0.82	0.7	0.86	0.88	0.91	0.88	**0.85**
AUC(*β*)	0.9	0.84	0.94	0.77	0.89	0.88	0.96	0.8	**0.87**
AUC(*γ*)	0.97	0.94	0.94	0.97	0.92	0.96	0.98	0.97	**0.96**
S1(*α*)	0.79 ± 0.05	1.02 ± 0.76	1.03 ± 0.35	0.71 ± 0.07	0.7 ± 0.03	0.8 ± 0.06	0.88 ± 0.37	0.74 ± 0.18	**0.83 ± 0.13**
S2(*α*)	0.61 ± 0.01	0.6 ± 0.03	0.66 ± 0.03	0.61 ± 0.01	0.5 ± 0.02	0.6 ± 0.04	0.61 ± 0.03	0.6 ± 0.03	**0.59 ± 0.04**
S1(*β*)	0.57 ± 0.15	0.63 ± 0.1	0.6 ± 0.1	0.65 ± 0.21	0.65 ± 0.08	0.63 ± 0.11	0.67 ± 0.11	0.56 ± 0.17	**0.62 ± 0.03**
S2(*β*)	0.29 ± 0.07	0.39 ± 0.12	0.26 ± 0.06	0.39 ± 0.14	0.4 ± 0.06	0.32 ± 0.13	0.32 ± 0.05	0.37 ± 0.11	**0.34 ± 0.05**
S1(*γ*)	2.44 ± 0.82	2.7 ± 0.96	2.74 ± 1.33	2.91 ± 0.85	3.33 ± 1.18	3.18 ± 1.2	2.56 ± 0.87	3.12 ± 1.05	**2.89 ± 1.07**
S2(*γ*)	7.47 ± 2.13	6.2 ± 1.92	6.74 ± 2.32	6.63 ± 1.6	7.02 ± 2.25	8.24 ± 2.35	7.8 ± 2.6	6.83 ± 1.49	**7 ± 2.5**
HR (bpm)	162	180	170	194	186	192	198	180	

**Table 2:** Significance values (Mann-Whitney *U* test), range (min and max), and the area under curve (AUC) results obtained for all subjects and for each proposed feature.

Feature	*P* value	Range (S1)	Range (S2)	AUC
*α*	<0.0001	0.5–2	0.5–0.92	0.85
*β*	<0.0001	0.3–1	0.13–0.88	0.87
*γ*	<0.0001	1–7.8	2–19.2	**0.96**

**Table 3:** AUC results for 3 levels of noise added on different sounds in the database.

Feature	10 dB	5 dB	0 dB
*α*	0.85	0.82	0.8
*β*	0.87	0.85	0.83
*γ*	0.96	0.93	0.91
HFS	0.6	0.58	0.57
